# The Daily Context of Mental and Cognitive Health: Using an Ecological Momentary Assessment (EMA) Approach

**DOI:** 10.1093/geroni/igaf122.460

**Published:** 2025-12-31

**Authors:** Jee eun Kang

## Abstract

Mental health and cognitive performance fluctuate with daily experiences, yet most research relies on retrospective reports that do not capture real-time variations. Ecological momentary assessment (EMA) enables examination of how contextual experiences unfold in everyday life. This symposium showcases studies using EMA to investigate how psychological and cognitive processes in daily life influence cognitive experiences in aging populations and how they represent potential entry points for intervention. First, Dr. Compernolle will describe social and physical contexts that contribute to fluctuations in loneliness—an important indicator of social and mental well-being—using findings from the Chicago Health and Activity Space in Real Time study. Next, Drs. Kang and Felt will highlight the dynamic relationship between loneliness and cognitive performance in daily life in an Einstein Aging Study cohort. Kang will discuss the reciprocal association between loneliness and daily memory lapses, demonstrating how loneliness both predicts and results from memory lapses. Felt will explore bidirectional links between loneliness and objective cognitive performance, showing that higher cognitive performance predicts lower subsequent loneliness, whereas loneliness is associated with slower processing speed. Finally, Dr. Sutin will describe how fluctuations in momentary purpose in life link with cognitive performance, revealing that while greater purpose is linked to better cognitive performance, it may also temporarily tax attentional resources, leading to increased memory lapses. Together, these presentations will highlight how momentary psychological experiences—including loneliness and purpose in life—shape cognitive performance and vice versa, emphasizing the importance of real-time assessments in understanding and promoting cognitive and emotional well-being in later life.

